# A 17-gene-expression profile to improve prognosis prediction in childhood acute myeloid leukemia

**DOI:** 10.18632/oncotarget.26116

**Published:** 2018-09-21

**Authors:** Nicolas Duployez, Claude Preudhomme, Meyling Cheok

**Affiliations:** Centre Hospitalier Regional Universitaire de Lille, Laboratory of Hematology, Biology and Pathology Center, Lille, France; INSERM, Universitaire de Lille, Jean-Pierre AUBERT Research Center, Lille, France

**Keywords:** childhood, acute myeloid leukemia, leukemic stem cell signature, prognostic

Pediatric acute myeloid leukemia (AML) is a molecularly and clinically heterogeneous disease accounting for about 20% of acute leukemia in children. Major advances have been made do better define the genetic basis of AML. Underlying cytogenetic and molecular aberrations are now routinely used to inform disease classification and stratify patients into favorable, unfavorable or intermediate risk categories, the latter encompassing about a half of patients [1]. Although outcome has greatly improved over the past decade, relapse occurs in about 30% of pediatric AML and represents the major cause of mortality. Notably, a large proportion of children with AML still relapse regardless of known poor risk factors.

Treatment failure and subsequent relapse have been attributed to the existence of leukemic stem cells (LSCs) harboring stemness properties including self-renewal capacities and resistance to current cytotoxic therapies [2]. However, specific identification of LSCs and their discrimination from normal hematopoietic stem cells is not fully established and LSC activity is assessed by their ability to generate leukemia in immunocompromised mice, limiting their use as a predictive tool in clinical activity. Recently, Ng et al. identified an optimized 17- gene expression signature for LSCs [3]. To do this, adult AML samples were divided into cell fractions based on CD34 and CD38 expressions and each fraction was transplanted into immunocompromised mice. Then, differential gene expression patterns between leukemia-initiating cell fractions and fractions that did not initiate leukemia allowed the identification of an optimal panel of 17 genes associated with stemness properties. The LSC17 score was shown to reliably differentiate patients into high and low risk groups in five independent cohorts of adult AML.

While pediatric and adult AML represent two genetically distinct diseases [4], we reasoned that pediatric and adult LSCs share common gene expression programs. In this respect, we investigated the LSC17 score as a prognostic tool in AML children treated in the French ELAM02 clinical trial [5]. Since the LSC17 score was initially established using adult AML samples with the exception of core binding-factor (CBF)-AML [3], we focused our analyses on 163 non-CBF AML patients. The 17-gene expression profile was determined in diagnostic AML samples by NanoString technology and the patients were then stratified by LSC17 quartile groups (lowest 25%, intermediate 50% and highest 25%). As demonstrated in cohorts of adult AML patients [3], pediatric AML with low LSC17 score showed a significant better outcome than pediatric AML with high score both in event-free survival and overall survival. These results were independently validated using the same criteria based on available TARGET-AML RNA-seq data of 171 non-CBF-pediatric AML [6]. Additionally, a high LSC17 score was associated with induction failure in the ELAM02 cohort whereas it was not statistically significant in the TARGET-AML cohort. The prognostic relevance of the LSC17 score was independent from usual diagnostic parameters including age, gender, percent of bone marrow blasts, white blood cell count at diagnosis and cytogenetics. However, among molecular aberrations, *CEBPA* double mutations and *NPM1* mutations without *FLT3*-internal tandem duplication were not found in patients with high LSC17 scores. By contrast, *RUNX1* mutations were associated with higher LSC17 scores in this cohort. Further studies are needed to extend those findings and determine whether the LSC17 score has any association with other molecular aberrations. Importantly, the LSC17 score retained a strong value in AML without favorable and adverse molecular-risk factors as previously defined by our group [1] as well as in cytogenetically normal-AML which makes up respectively 50% and 25% of all pediatric AML. This is critical in routine practice since these AML categories have been shown to display heterogeneous outcome. Finally, when known prognostic factors were included in the multivariate analysis, a high LSC17 score remained the strongest prognostic marker in our cohort.

In conclusion, we extended the applicability of the LSC17 score from adult AML to childhood AML. Our data suggest that the LSC17 score is a determinant of chemotherapy resistance and relapse in pediatric AML. Consequently, future studies should investigate interactions between the LSC17 signature and measurable residual disease routinely used to assess treatment efficiency and follow-up in AML patients. Furthermore, since the LSC17 score has been shown to predict response to gemtuzumab ozogamicin (GO) in adult AML patients, when added to standard chemotherapy, this fact has to be assessed in childhood AML [3, 7]. Current technologies now allow the rapid and reliable generation of gene-expression data to evaluate LSC activity for clinical care. How to precisely implement these findings for patients with AML is a challenge for the next years to come.
